# ZIF-L to ZIF-8 Transformation: Morphology and Structure Controls

**DOI:** 10.3390/nano12234224

**Published:** 2022-11-27

**Authors:** Chanjong Yu, Young Jae Kim, Jongbum Kim, Kiwon Eum

**Affiliations:** School of Chemical Engineering, Soongsil University, Seoul 06978, Republic of Korea

**Keywords:** ZIF-L, ZIF-8, morphology control, metal-organic frameworks

## Abstract

The control of the structure, shape, and components of metal-organic frameworks, in which metal ions and organic ligands coordinate to form crystalline nanopore structures, plays an important role in the use of many electrochemical applications, such as energy storage, high-performance photovoltaic devices, and supercapacitors. In this study, systematic controls of synthesis variables were performed to control the morphology of ZIF-8 during the ZIF-L-to-ZIF-8 transformation of ZIF-L, which has the same building block as ZIF-8 but forms a two-dimensional structure. Furthermore, additional precursors or surfactants (Zn^2+^, 2mIm, and CTAB) were introduced during the transition to determine whether the alteration could be regulated. Lastly, the partial substitution insertion of a new organic precursor, 2abIm, during the ZIF-L-to-ZIF-8 transformation of ZIF-L was achieved, and modulation of the adsorption and pore characteristics (suppression of gate-opening properties of ZIF-8) has been confirmed.

## 1. Introduction

Metal-organic frameworks, which combine metal ions with organic ligands to form a crystalline nanopore structure, have the advantage of tuning features (e.g., pore size, adsorption capabilities, etc.) to be controlled by combining different metals and organic precursors [1,2]. Thus, it is recognized as a material having significant promise for usage in a wide range of applications, including gas storage [3,4], separation [5,6,7], catalysis [8], and sensing [9]. Furthermore, metal-organic frameworks are good starting materials for the creation of valuable metal oxides and carbon compounds for use in electrochemical applications, including energy storage [10], photovoltaic devices [11], and supercapacitors [12]. The component and structural elements of these metal-organic frameworks usually define their qualities, but the morphological features of metal-organic frameworks also play an essential role in fine-tuning their properties [13].

For example, controlling the shape of the metal-organic framework to increase the external surface area of a certain crystal facet, can influence the exposure of active sites that affect chemical reaction barriers and, therefore, affect the electrochemical catalytic activity [14]. A high-performance polycrystalline membrane can be manufactured by preventing the deep penetration of solvents during membrane fabrication, via a 2D-structured seed crystal layer approach [15]. As a result, controlling the creation of the metal-organic framework in terms of structure, shape, and composition is regarded as the most critical component in building a well-designed metal-organic framework with acceptable attributes. Numerous investigations have attempted to regulate the morphology of MOFs by tuning the synthesis condition. (e.g., However, there is no technique that can be broadly applied to metal-organic frameworks, and there is a fundamental lack of understanding of inclusion of additives [3], pH control [16], and synthesis solvent control [17]). how to stimulate the formation of crystals with a target shape.

A recent study demonstrates that ZIF-L undergoes ZIF-L-to-ZIF-8 phase transformation into ZIF-8 when exposed to a high-temperature organic solvent [18,19,20,21]. ZIF-8, which has received the most attention due to its high thermal and chemical stability, as well as its stable and versatile pore properties, is made up of a coordination bond between Zn^2+^ and 2mIm, and is found in the form of sodalite (SOD) with a pore cavity of 11.6 Å, accessible through the theoretical pore aperture of 3.4 Å [22]. ZIF-L, on the other hand, has the same secondary building unit and components as ZIF-8, but has a two-dimensional structure. The parallel layers form hydrogen bonds (metastable) between 2mIm and 2mIm of monodentate and 3.97 Å apart [23]. In this study, the systematic control of synthesis parameters was employed to regulate ZIF-8’s morphology during the ZIF-L-to-ZIF-8 transformation of ZIF-L. During the transition, Zn^2+^, 2mIm, and CTAB are introduced to determine whether the alteration can be modulated. Lastly, the substitutional insertion of secondary organic precursors is confirmed during the ZIF-L-to-ZIF-8 structural conversion, and when the modulation of the adsorption and pore characteristics has been confirmed.

## 2. Materials and Methods

### 2.1. Materials

2-methylimidazole (99%, 2mIm), zinc nitrate hexahydrate (98%, Zn(NO_3_)_2_ ∙ 6H_2_O), 2-aminobenzimidazole (97%, 2abIm), and Cetyltrimethylammonium bromide (CTAP) were obtained from Sigma-Aldrich Korea (Seoul, Republic of Korea). N,N-Dimethylacetamide (99.5%, DMAc), was purchased from DaeJong-Chem (Seoul, Republic of Korea). N,N-Dimethylformamide (99.9%, DMF) was purchased from SamChun-Chem (Seoul, Republic of Korea). Homemade deionized water (DI-water) was used for the synthesis (Rephile Bioscience, Seoul, Republic of Korea).

### 2.2. ZIF-L Crystals Synthesis

Zn(NO_3_)_2_∙6H_2_O was dissolved in 9.6 mL DI-water and 2mIm was dissolved in 28 mL DI-water, respectively. The amount of Zn(NO_3_)_2_∙6H_2_O and 2mIm was calculated to represent the specific molar ratio (e.g., 0.28 g of Zn(NO_3_)_2_∙6H_2_O, and 0.77 g of 2mIm for Zn^2+^:2mIm:H_2_O ratio of 1:10:2222). Then, 28 ml of 2mIm aqueous solution was added dropwise to the zinc nitrate aqueous solution while stirring. Finally, the cloudy solution was stirred for 2 h at room temperature and centrifuged at 11,000 rpm for 40 min. As-made ZIF-L powders were washed with DI-water three times and then dried in a vacuum oven at 80 °C for 12 h.

### 2.3. ZIF-LtoZIF-8 Transformation

A total of 0.08 g of ZIF-L crystal was dispersed in 8 mL DMAc/H_2_O (2:1, 4:1, and 8:1 *v*/*v*%, respectively) solution and sonicated for 1 min. Dispersion was confirmed by observing cloud solution without precipitate. Then, the vial was placed in a 353 K preheated oven for 8 h, followed by natural cool down. The resulting crystals were washed with DI-water three times, followed by vacuum degassing at 353 K for 12 h. For additional organic and inorganic precursors or surfactants solution, the below was used instead of pure DMAc/H_2_O solution.

1. Addition of 1 M Zn

A total of 2.38 g of Zn(NO_3_)_2_·6H_2_O was dissolved in 8 mL of DMAc/H_2_O (2:1 v%), and then 0.08 g of ZIF-L crystal was dispersed and sonicated for 1 min. The sequential procedure is the same as described above.

2. Addition of 0.1 M 2mIm

A total of 0.066 g of 2mIm was dissolved in 8 mL of DMAc/H_2_O (2:1 v%) and then, 0.08 g of ZIF-L crystal was dispersed and sonicated for 1 min. The sequential procedure is the same as described above.

3. Addition of 0.01–0.05 M CTAB

A total of 0.029–0.145 g of CTAB was dissolved in 8 mL of DMAc/H_2_O (2:1 v%), and then 0.08 g of ZIF-L crystal was dispersed and sonicated for 1 min. The sequential procedure is the same as described above.

4. Addition of 0.1–0.5 M 2abIm

A total of 0.11–0.55 g of 2abIm was dissolved in 8 mL of DMAc/H_2_O (2:1 v%) solution, and then 0.08 g of ZIF-L crystal was dispersed and sonicated for 1 min. The sequential procedure is the same as described above, except for the oven temperature (393 K).

### 2.4. Characterization

X-ray diffraction patterns were obtained from a Bruker D2 Phaser diffractometer (Billerica, MA, USA) at ambient temperature using Cu Kα radiation of λ = 0.154 nm and a scanning range of 5−40° 2θ. The crystal SEM images were collected with ZEISS GeminiSEM 300 (Oberkochen, Germany). All samples were pretreated and sputter coated with Pt (Q150R Plus-Rotary Pumped Coater, East Sussex, UK). The N_2_ physisorption isotherms were obtained from a Micromeritics ASAP 2020 (Norcross, GA, USA) surface area analyzer at 77 K. All samples were properly degassed at 393 K for 12 h prior to their use. ATR-FTIR was performed with a Nicollet spectrometer (Waltham, MA, USA) using a DTGS detector. Liquid ^1^H-NMR spectra were collected with a JEOL 600 MHz NMR (Peabody, MA, USA) using 64 scans.

## 3. Results

**Figure 1:**
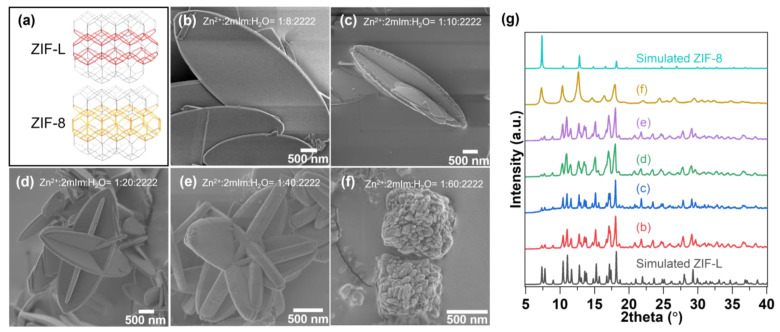
(**a**) Crystal structure of ZIF-L and ZIF-8, SEM images of ZIF-L varying linker ratio (**b**) Zn^2+^:2mIm:H_2_O = 1:8:2222, (**c**) Zn^2+^:2mIm:H_2_O = 1:10:2222, (**d**) Zn^2+^:2mIm:H_2_O = 1:20:2222, (**e**) Zn^2+^:2mIm:H_2_O = 1:40:2222, (**f**) Zn^2+^:2mIm:H_2_O = 1:60:2222, (**g**) XRD pattern of ZIFs crystals as illustrated in (**b**–**f**).

ZIF-L features plate-like crystallinity with a high aspect ratio due to its anisotropic pore system shown in Figure 1a, and the morphology can be tuned by controlling the synthesis condition. A scanning electron microscopy (SEM) image of leaf-shaped ZIF-L (Zn^2+^:2mIm:H_2_O = 1:8:2222 ratio, (1:8:2222 later)) is shown in Figure 1b, and crystal structure was confirmed by XRD (Figure 1g). When increasing the ratio of the organic precursor (2mIm) while keeping the ratio of the rest component (1:10:2222), crystal growth in the direction perpendicular to the (001) crystal facet is promoted, as illustrated in Figure 1c. As the ratio of the organic precursor further increased (Figure 1c–e), a more vertical cross-linked leaf-shaped crystal formation was observed on the ZIF-L surface, and the thickness of the crystal gradually increased from 110 nm to 253 nm. Based on the observation, it can be inferred that excessive unreacted 2mIm precursor was attracted to monodentate 2mIm terminal group on (001) facet, increasing the probability of vertical growth perpendicular to the (001) plane. The increase in ZIF-L sheet thickness also supports the hypothesis mentioned above. When the ratio of 2mIm to Zn^2+^ exceeded 60, formation of spherical ZIF-8 crystal with rough surface texture (Figure 1f,g) was confirmed. As stated previously, ZIF-L is metastable due to the existence of unterminated coordination bonds. Thus, it can be used as a starting material for synthesizing a ZIF-8 that has a similar structure (Figure 1a) via ZIF-L-to-ZIF-8 transformation [17]. Here, leaf-shaped ZIF-L particles (Figure 1b) were exposed to a 333K pure solvent containing a 2:1 (*v*/*v*) DMAc/H_2_O mixture.

**Figure 2:**
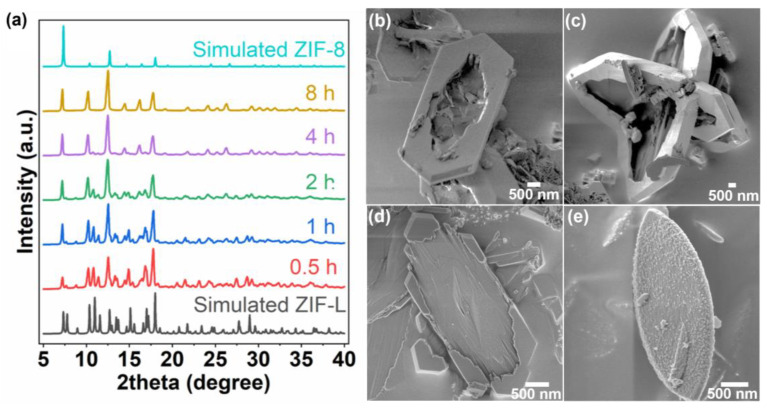
(**a**) XRD patterns of ZIF-L crystals as a function of exposure time (0.5, 1, 2, 4, and 8 h) in a pure solvent (2:1 (*v*/*v*) DMAc/H_2_O) at 333 K, SEM images of (**b**) leaf-shaped, and (**c**) cross-leaf shaped ZIF-L after the pure solvent (DMAc/H_2_O = 2:1 *v*/*v*) exposure, SEM images of leaf-shaped ZIF-L upon 8 h exposure to (**d**) 1:8 (*v*/*v*) DMAc/H_2_O, and (**e**) 8:1 (*v*/*v*) DMAc/H_2_O solvent.

The gradual ZIF-L to ZIF-8 transition was confirmed by XRD (Figure 2a) as the solvent exposure time increased, and complete transformation was observed after 8 h of synthesis. Interestingly, the resulting crystal shows a center-etched ring-shaped morphology, shown in Figure 2b. Under the identical reaction conditions, the cross-linked ZIF-L crystal (Figure 1c) was likewise transformed into a hollow cross-leaf shape (Figure 2c). It is because the CH_3_COOH (AcOH) and (CH_3_)_2_NH (d-MA), generated by the reaction of DMAc and water, are thought to be the sources of both structural and morphological alteration. It is noted that the above ZIF-L to ZIF-8 transformation can be accomplished with environmentally friendly solvents [18,20] or even without solvents [21]; however, a DMAc/DI-water solvent system can selectively etch the (001) facet of the ZIF-L, thereby yielding ZIF-8 with ring or hollow-cross leaf morphology. The interlayer bonding of ZIF-L is relatively unstable due to weak hydrogen bonding between 2mIm and monodentate 2mIm. AcOH, a weak acid, has the ability to dissolve the coordination bond between Zn^2+^ and 2mIm, and d-MA could promote the dehydrogenation of monodentate 2mIm, leading to the preferential etching of the (001) plane. As the dissociation of metal and organic precursor ions develops over time, the local concentration of precursor ions around the ZIF-L crystals becomes sufficient for the formation of coordination bonds toward ZIF-8. The bond formation is thought to be focused around the (010) ring, where etching is relatively slow, creating the ring-shaped ZIF-8 structure. To validate our hypothesis, ZIF-L was subjected to the same conditions (333 K, 8 h) in pure H_2_O or DMAc solvents, and neither a morphological nor structural alteration was observed. Additionally, the pH of the solvent was adjusted by altering the ratio of DMAc to H_2_O, and the influence on the structure and/or morphology change of ZIF-L to ZIF-8 was examined. The pH measurement results, as the DMAc/H_2_O volume ratio changed, were tabulated in Table 1. The pH of the 2:1 (*v*/*v*) DMAc/H_2_O solvent was approximately 10.2, and as the DMAc/H_2_O ratio was decreased, the pH gradually declined (AcOH increased). As the pH decreased, the etching of the crystal extended not only to the (001) plane but also to the periphery of the ring, and the edges of the crystal were fractured (Figure 2d). On the other hand, as the pH gradually increased, the etching of the crystal gradually slowed down. As illustrated in Figure 2e, the resulting ZIF-L, synthesized from 8:1 (*v*/*v*) DMAc/H_2_O solvent, was found to have a rough texture on the (001) surface; however, no structural conversion to ZIF-8 was confirmed.

**Figure 3:**
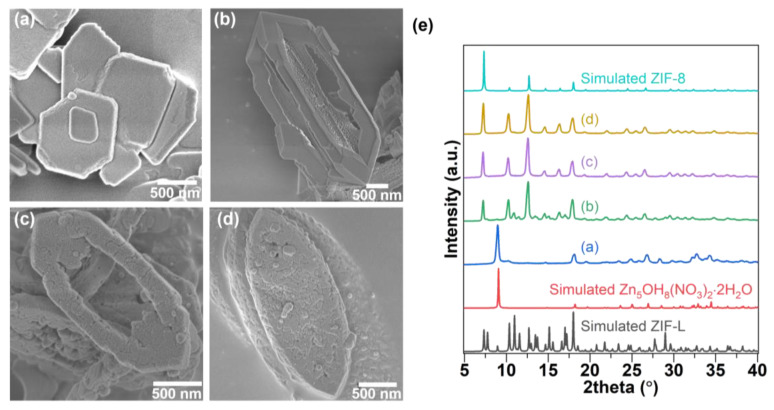
SEM images of ZIF-L after 8 h of exposure with (**a**) 1 M Zn^2+^, (**b**) 0.1 M 2mIm, (**c**) 0.01 M CTAB, and (**d**) 0.05 M CTAB containing DMAc/H_2_O solution at 333 K, (**e**) XRD pattern of crystals as illustrated in (**a**–**d**).

As previously stated, ZIF-L undergoes a phase transition to ZIF-8 when exposed to a DMAc/H_2_O solution. During the transition, additional precursors or surfactants (Zn^2+^, 2mIm, and CTAB) were introduced to examine if the morphological change can be modulated. As shown in Figure 3a,e, a layered zinc hydroxy in the form of thin plates, with a thickness of about 100 nm, were generated when 1 M Zn^2+^ ions were added into the 2:1 (*v*/*v*) DMAc/H_2_O solution. This is because the Zn(NO_3_)_2_·6H_2_O contained in the precursor solution can provide the -NO^3−^, Zn^2+^, and -OH groups necessary for the formation of layered zinc hydroxy. When the 0.1 M 2mIm organic precursor was added, the structural change and internal etching of ZIF-L were significantly suppressed, thereby maintaining the ZIF-L structure (Figure 3b,e). Deacon et al. reported a similar inhibition of the ZIF-L to ZIF-8 transformation [24]. It can be inferred that the high 2mIm concentration gradient in the solution phase could prevent the de-coordination of monodentate 2mIm from the ZIF-L surface. Finally, the effect of surfactant CTAB on the phase transition was examined. As shown in Figure 3c–e, the degree of internal etching during the phase transition can be controlled via tuning the CTAB concentration in the solution. The rough surface texture on the (001) facet was observed. This is because CTAB is a surfactant and can facilitate the aggregation of dissociated precursor ions on the surface of the ZIF-L plane; thus, etching could be prevented during the bond reconstruction.

**Figure 4:**
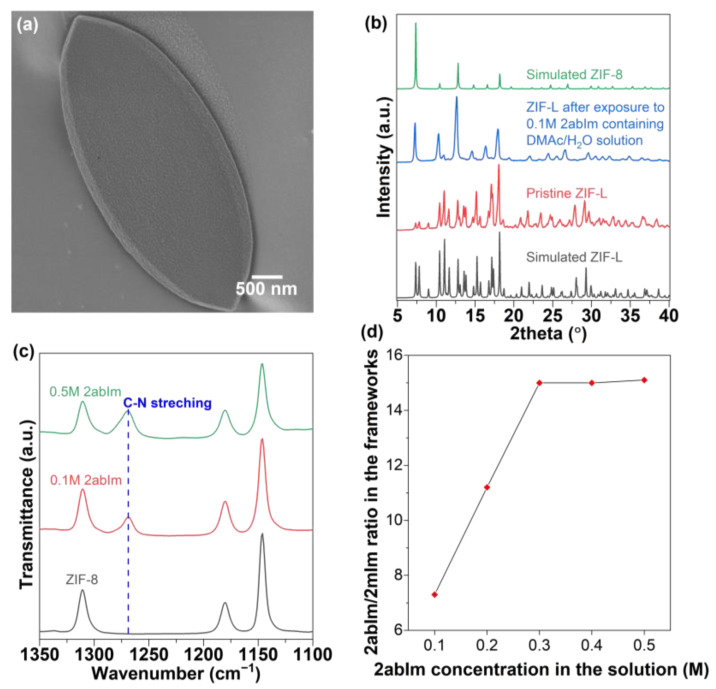
(**a**) SEM image of ZIF-L upon exposure to 0.1 M 2abIm containing 2:1 (*v*/*v*) DMAc/H_2_O solution for 8 h at 373 K, (**b**) XRD patterns of ZIF-L before and after exposure to 0.1 M 2abIm containing 2:1 (*v*/*v*) DMAc/H_2_O solution for 8 h at 373 K, (**c**) ATR-FTIR on 0.1 M, and 0.5 M of 2abIm treated ZIF-8 crystals, (**d**) composition analysis curves of 2abIm-treated ZIF-8 crystals obtained by solution ^1^H-NMR.

Finally, an imidazole-based organic precursor that is capable of coordinating with Zn^2+^ was added during the structural alteration from ZIF-L to ZIF-8. A Leaf-shaped ZIF-L crystal was exposed to 2abIm containing a 2:1 (*v*/*v*) DMAc:H_2_O solution at 373 K for 8 h. As seen in Figure 4a,b, the SOD topology of ZIF-8 was confirmed, however, no particular morphology change was observed. Figure 4c shows the 1100–1350 cm^−1^ range of attenuated total reflectance-Fourier transform infrared (ATR-FTIR) spectra of ZIF-8 crystals, before and after the treatment. The full spectrum of collected ATR-FTIR spectra is available in the supporting information (Figure S1). The incorporation of 2abIm in the ZIF-8 crystals is supported by the appearance of a band near 1270 cm^−1^ that is not observed in pure ZIF-8 crystals. According to the literature [25], the adsorption at 1270 cm^−1^ is due to the C−N stretching vibrations of 2abIm. As the concentration of 2abIm in the synthesis solution increased, a gradual elevation in the 1270 cm^−1^ peak was also observed, indicating an increase in the 2abIm linker amount inside the frameworks. Consistently, solution-phase ^1^H-NMR spectroscopy of 2abIm-treated ZIF-8 crystals (later 2abIm-ZIF-8) reveals the existence of 2abIm with 2mIm (Figure S2). Additionally, a “composition curve” was prepared to determine the appropriate 2abIm concentration for a particular 2abIm/2mIm ratio ZIFs crystal (Figure 4d). The maximum 2abIm/2mIm ratio that can be achieved via ZIF-L to ZIF-8 transformation is about 15%.

**Figure 5:**
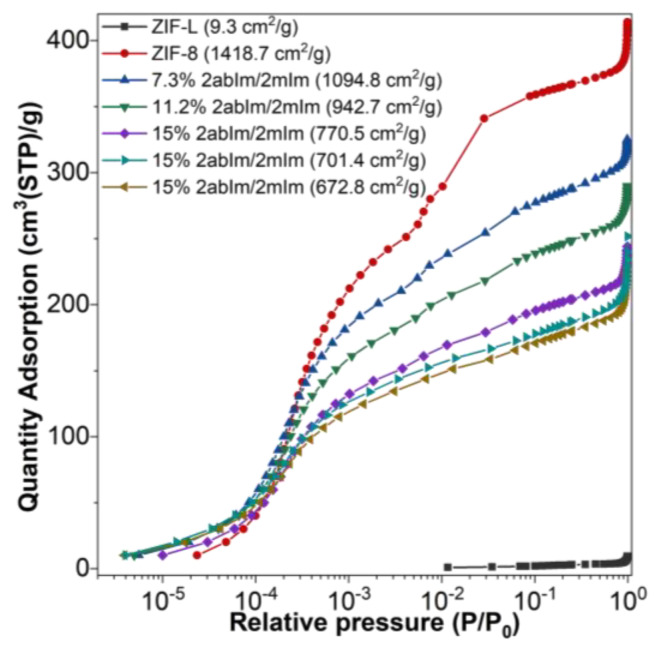
N_2_ adsorption isotherms of ZIF-L, ZIF-L-derived ZIF-8, and 2abIm-ZIF-8 varying 2abIm/2mIm ratios.

An isothermal nitrogen adsorption examination (Figure 5) was conducted to investigate the adsorption properties of the ZIF-L, ZIF-L-derived ZIF-8, and 2abIm-ZIF-8. The conversion from ZIF-L to ZIF-8 resulted in a significant increment in specific surface area and no significant difference was observed, compared to the in situ ZIF-8 crystal. It is noted that a large amount of N_2_ adsorption was confirmed even at the extremely low relative pressure of 5 × 10^−4^ P/P_0,_ due to the microporous nature of ZIF-8. In addition, the second adsorption occurs near 5 × 10^−3^ P/P_0_, due to the pressure-induced pore reconstruction (gate-opening effect) [26]. As nitrogen is adsorbed on the ZIF-8 surface, the Zn-N bond of ZIF-8 rotates, creating a new adsorption site. This is due to the flexibility of intermolecular chains, which is not observed in inorganic materials like zeolite. In the case of ZIF-L-derived 2abIm-ZIF-8, a dramatic reduction in the gate-opening phenomenon (decreased frameworks flexibility) was observed, along with the gradual decrement in specific surface area in proportion to the 2abIm ratio. It is because the bulky 2abIm benzene ring induces steric hindrance inside the structure, consequently restricting molecular bond rotation. These controllable gate-opening properties are beneficial to various applications. For example, gate-opening exhibits favorable adsorption properties, such as an increase in adsorption capacity [27]. However, in a diffusion-selective membrane used for molecular separation, it may impede the membrane’s ability to demonstrate sharp separation performance [28]. In this case, we can suppress the gate-opening effect by simply adding 2abIm into the frameworks during the transformation.

## 4. Conclusions

The regulation of ZIF-L morphology, from leaf to cross-linked leaf, was achieved by adjusting the ratio of organic precursors in a synthesis solution. Then, crystal conversion from ZIF-L to ZIF-8 was conducted by exposing the 2:1 (*v*/*v*) DMAc/H_2_O solvent at a mild synthesis condition (333 K, 1 atm). During the transformation, preferential etching toward the (001) facet of ZIF-L was induced by the AcOH and d-MA (formed by the reaction of DMAc and water), thereby forming the ring or hollow-cross leaf-shaped ZIF-8 crystals. Furthermore, it was investigated whether the alteration in crystal morphology might be affected by the introduction of additional organic or inorganic precursors or surfactants (Zn^2+^, 2mIm, and CTAB). The addition of Zn^2+^ ions produced layered zinc hydroxy with a thickness of 100 nm. CTAB aided the surface aggregation of dissociated ions, indicating that the degree of internal etching can be modulated. Finally, it was conceivable to produce a mixed precursor ZIF-8 crystal during the structural transition from ZIF-L to ZIF-8 by adding a secondary ligand. The maximum 2abIm/2mIm ratio achievable via ZIF-L to ZIF-8 transformation is about 15%, and a gradual decrease in BET-specific surface area, from 1094 cm^2^/g to 673 cm^2^/g, was observed as the 2abIm/2mIm ratio decreased. Importantly, modulation of pore characteristics has been confirmed by successfully reducing the gate-opening effect of ZIF-8.

## Figures and Tables

**Table 1:** Summary of pH as a function of the DMAc/H_2_O ratio.

DMAc:H_2_O Ratio	pH
1:1	9.7
1:2	7.5
1:4	7.1
1:8	6.6
2:1	10.2
4:1	10.1
8:1	11.2

## Data Availability

The data used to support the findings of this study are available from the corresponding author upon request.

## Supplementary Materials

The following supporting information can be downloaded at: https://www.mdpi.com/article/10.3390/nano12234224/s1, Figure S1. Full range ATR-FTIR spectra of ZIF-L-derived ZIF-8, and 0.1–0.5 M abIm added ZIF-8 crystals, Figure S2. ^1^H-NMR spectra of (a) DMSO-d6/DCl Solvent (black curve) δ 2.96 (s, 3H), δ 6.95 (s, 1H), ZIF-8 in DMSO-d6/DCl (red curve) δ 7.45 (m, 2H), δ 7.09 (s, 1H), δ 2.96 (s, 3H), and 0.1–0.5 M 2abIm ZIF-8 in DMSO-d6/DCl (blue and green curve) δ 7.45 (m, 2H), δ 7.32 (m, 2H), δ 7.15 (m, 2H), δ 7.09 (s, 1H), δ 2.96 (s, 3H). (b) ^1^H-NMR spectra of expanded range δ 7–7.5.
