# Supplementary material for: ZIF-L to ZIF-8 Transformation: Morphology and Structure Controls
Source: Nanomaterials (Basel). 2022 Nov 27;12(23):4224. doi: 10.3390/nano12234224 (PMC9740542; doi:10.3390/nano12234224)
Supplement: Supplementary file 1 [file nanomaterials-12-04224-s001.zip › nanomaterials-2056656-supplementary.pdf]

Supplementary Materials

ZIF-L to ZIF-8 Transformation: Morphology and Structure Controls

Chanjong Yu, Young Jae Kim, Jongbum Kim and Kiwon Eum *

School of Chemical Engineering, Soongsil University, Seoul 06978, Republic of Korea
* Correspondence: kiwon.eum@ssu.ac.kr

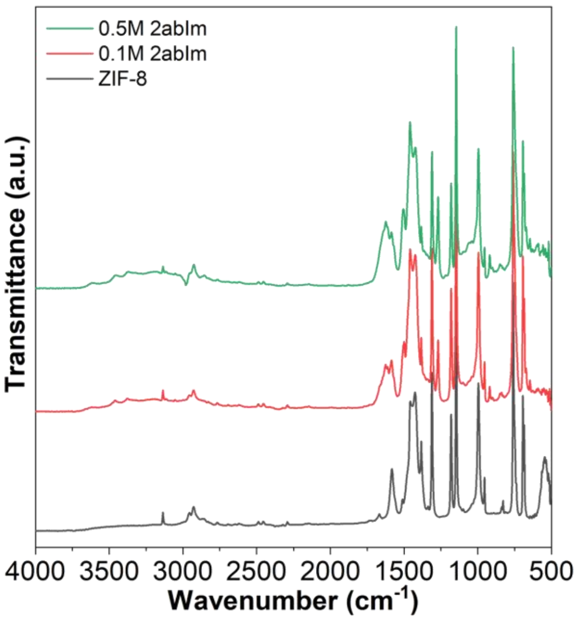

Figure S1. Full range ATR-FTIR on 0.1 M, and 0.5 M of 2abIm treated ZIF-8 crystals.

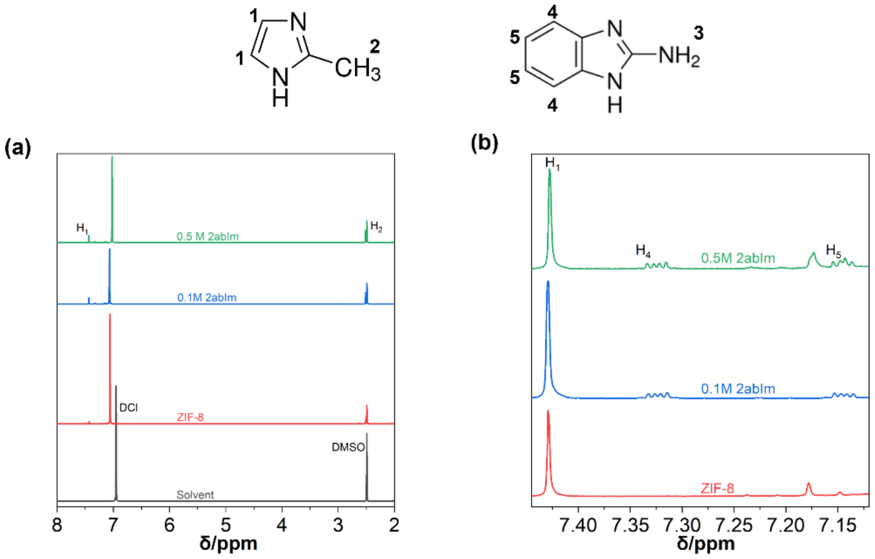

Figure S2. ^{1}H-NMR spectra of (**a**) DMSO-*d6*/DCl Solvent (black curve) δ 2.96 (s, 3H), δ 6.95 (s, 1H), ZIF-8 in DMSO-*d6*/DCl (red curve) δ 7.45 (m, 2H), δ 7.09 (s, 1H), δ 2.96 (s, 3H), and 0.1M-0.5M 2abIm ZIF-8 in DMSO-d6/DCl (blue and green curve) δ 7.45 (m, 2H), δ 7.32 (m, 2H), δ 7.15 (m, 2H), δ 7.09 (s, 1H), δ 2.96 (s, 3H). (b) 1H-NMR spectra of expaned range δ 7-7.5.
